# Motivations Influencing the Surgeon’s Healthcare Unit Choice to Perform Surgery: A Confirmatory Study in Portugal

**DOI:** 10.3390/ejihpe10010013

**Published:** 2019-10-24

**Authors:** Diana Nora, Diogo Guedes Vidal, Lilian Monteiro Ferrari Viterbo, Maria Alzira Pimenta Dinis, Hélder Fernando Pedrosa Sousa

**Affiliations:** 1 MBA-Advanced Course in Healthcare Organizations and Services, University Fernando Pessoa (UFP), Praça 9 de Abril 349, 4249-004 Porto, Portugal; dnora@ufp.edu.pt; 2 UFP Energy, Environment and Health Research Unit (FP-ENAS), University Fernando Pessoa (UFP), Praça 9 de Abril 349, 4249-004 Porto, Portugal; lilianmferrari@gmail.com (L.M.F.V.); madinis@ufp.edu.pt (M.A.P.D.); 3 Department of Mathematics (DM. UTAD), University of Trás-os-Montes and Alto Douro, Quinta de Prados, 5001-801 Vila Real, Portugal; hfps@utad.pt

**Keywords:** surgical practice, surgeons’ motivation, operating room (OR)

## Abstract

Quality surgical practice is a fundamental subject in health institutions, and it is important to understand the structural and organizational conditions of the operating room (OR). The present exploratory study sought to understand the motivations that underlie the choice of surgeons for the best healthcare unit to perform surgery, as well as the characteristics of those professionals regarding age, years of work experience, and sex. A questionnaire survey was administered to a convenience sample of 99 surgeons, 67.3% male and 32.7% female, aged 37 to 66 (*M* = 23.7; *Std* = 8.92). The results show that at the top of the surgeons’ motivations to perform surgery is the 77.8% attributed to the human resources and equipment available and at the other extreme the 3% to the previous online visit to facilities. This study opens important clues to the development of more in-depth and comparative approaches, necessary for the continuous improvement of the healthcare provided in the context of surgical practice.

## 1. Introduction

Health in Portugal has evolved positively in the last 40 years [1]. Changes in the living conditions of most Portuguese citizens in terms of basic sanitation, housing, and food have achieved significant gains, improving the population’s health indicators [2,3]. In addition, scientific and technological advances and the development and expansion of a universal and trend-free National Health Service have resulted in a significant improvement in healthcare delivery [4], although recent studies point to inequalities in access to quality healthcare [5,6,7].

The Portuguese National Health Service (NHS) has been reformed since its inception in 1979 by Law No. 56/79 of 15 September [8]. Based on a chronology of the evolution of the NHS by Vidal et al. [6], it can be stated that it was configured in six major periods:

▪ From 1970 to 1982, it comprised the creation and implementation of the NHS with the dissemination of primary healthcare;
▪ Between 1982 and 1995, the boundaries between public and private health services changed by continually improving the quality of service and human resources, through the access to European funds;
▪ The period from 1995 to 2002 was characterized by the increase of the public offer over the private one;
▪ The change from the existing model of health system to the NHS occurred between 2002 and 2005 with the stabilization of the public and private publics, namely with the creation of public–private partnerships;
▪ The implementation of public health policies, with the objective of modernizing and expanding the NHS, occurs between 2005 and 2009;
▪ Since 2010, the NHS has been the target of several economic cuts due to the crisis that Portugal went through and is still in a recovery phase today. Hospital reality has also been the subject of profound changes that accompany NHS trends. At a time when the need for healthcare integration has become even more evident, it is clear that hospitals have to follow this trend. The mission of hospitals requires that the activity of each healthcare unit has a special focus on its primary objective: To serve the patient in the most qualified, fastest, most effective, and most humane way, as stated by Ribeiro [9].

Studies from Fidler [10], Maarse [11], and Chapman [12] report that the private sector is becoming increasingly important in healthcare services in occidental countries and Portugal is no exception. In recent years, there has been a development and growth of private healthcare institutions, namely hospitals, clinics, palliative centers, and laboratories, which demonstrate the current propensity growth of the sector without the public part is able to fully meet global needs of the population [13,14,15].

In the hospital sector, the operating room (OR) is the focal point of numerous hospital activities and is directly or indirectly linked with most medical services and specialties, so a smooth functioning of structural conditions requires quality and motivated human and technical resources [16,17,18,19]. The investment in a modern OR is an imperative that can contribute to attracting surgeons and other healthcare professionals [20] whose highly differentiated performances can contribute to the excellence of these units. 

Portuguese surgeons reflect the reality experienced in the organization and practice of surgery worldwide. The multiple tasks they must perform do not always leave the time or energy to reflect on issues of their specialty. At the same time, there is a real revolution in the field of general surgery with the creation of sub-specialization sectors that, in the end, have even originated new surgical specialties. This has led to the development of new and sophisticated technical progress and altered diagnostic and therapeutic strategies in numerous areas. All of these changes created enormous difficulties for the old hospital structure, which increased the number of beds and subspecialties without the indispensable support structures evolving in parallel [19].

OR management is therefore an essential aspect of any modern hospital policy. The profound technological advances make it necessary for substantial equipment and organizational adjustments that do not, however, allow one to neglect knowing the opinions of different professionals in order to be able to design constructive proposals. 

Having presented the problematic of the conditions for a differentiated surgical practice, the starting point for this investigation is based on the following question: What are the motivations underlying the surgeons’ choice of the healthcare unit to perform surgery? Accordingly, this study aims to know and analyze the main motivations that lead Portuguese surgeons to choose where to perform surgery, intending to contribute not only to the improvement of the quality and performance of the healthcare services provided, but also to the entire development strategy of healthcare institutions, considering this study as a starting point for broader and more focused studies in the subject.

## 2. Literature Review

OR organization involves a multidisciplinary team covering specialties from different sectors [21,22]. The work in the central OR encompasses scheduled or urgent surgeries, and there is a significant increase in ambulatory surgery that justifies appropriate facilities. This guidance makes a powerful contribution to cost containment without compromising clinical quality, with very positive repercussions either at postoperative level or in the attempts to reduce waiting lists. It is a sector with great technical demands, both in terms of structures and procedures, as in terms of the healthcare professionals who work in it, thus assuming a huge relevance in the hospital where it is inserted. It is the primary target of organizational development and the containment of hospital expenses, seeking maximum use of the installed capacity and the resources involved in it [19].

Within the hospital context, OR has a particular emphasis since the quality and the level of results obtained by the surgical services are vital in terms of hospital environment, also because of the particular role that OR has in this specific clinical context. OR activity has a huge impact on the institution by the volume of important interactions with other sectors [23]. The OR is highly interdependent with intensive healthcare and more generally with the entire hospital environment. There are different types of intensive healthcare and their harmonious functioning is an essential basis for the flow of patients to be as adequate as possible [19]. 

The mobility of surgeons, as well as the main motivations for their allocation in a particular healthcare institution, is dependent on a considerable number of variables and their characterization is a challenge that many institutions seek to overcome. The Portuguese Medical Association [24] has made available a questionnaire for physicians in which various components influencing mobility and fixation, are considered.

The consulted literature does not allow one to obtain data that can accurately characterize the fixation of physicians. With regard to surgeons, and according to Marques et al. [25], it is not risky to predict that the existence of modern structures and quality organizational levels may be reasons to consider in the decision process to choose the best healthcare unit to perform surgery. The need to create referral health units requiring a concentration of resources and professionals is crucial to achieve excellence. This has been an intention subverted by an anarchic proliferation of these institutions, causing significant difficulties in the implementation of an clear surgeons fixation policy [26]. According to studies the from the Portuguese Ministry of Health [27] and Nora and Vidal [28,29], knowing in depth and framing the degrees of satisfaction of the surgeons with the working conditions available to healthcare professionals is an effective way in a surgeon’s allocation policy to a specific hospital location. Table 1 summarizes the latest studies on efficient OR management.

The current state-of-the-art literature on efficient OR management reveals a number of investigations on the subject under analysis that addresses distinct concerns, but with a common purpose: Optimization and satisfaction within medical-surgical practice. Time management in a surgery context is a major issue to which some researchers have given special attention. As an example, a study in China by Yang et al. [35] sought to develop a method for scheduling surgery that considers the surgeon preferences. This method application has resulted in a significant improvement in surgeon satisfaction given the efficiency of resource management.

In Portugal, the priority focus of reducing the waiting list for surgery was also the subject of reflection with the attempt to improve the surgical programming of a hospital through genetic heuristic processes [25]. The need for a detailed assessment of the OR practice is added to the desire to optimize it, namely through the development of algorithm models [36,38]. OR’s performance can benefit if there is previous recognition of the patients’ needs, a measure that is currently failing [37,39].

In addition to time management, the entire OR organization is currently a key issue. The increased supply of providers, the number of surgeries, and the need to respond quickly and effectively leads healthcare organizations to create strategies that address these issues. A California study by Montgomery and Schneller [32] sought to organize the selection of physician preference items over providers, resulting in the need to converge physician interests with hospital interests. The importance of information and communication techniques (ICTs) in the OR management and organization process has been discussed in a United States study by Plasters et al. [30], which concluded that ICTs provide only a fraction of the information needed to properly manage changing OR programming.

At the management and assessment level of the OR practice, the study of Marjamaa and Kirvela [31], sought to know the reality to propose management redefinition measures, namely to identify basal axes mentioned by surgeons. The collaboration and communication strategies among healthcare professionals were identified, considering an interdisciplinary logic as the most important dimension in differentiated clinical practice.

The reform of the current teaching models of health professionals, namely physicians/surgeons, is a field of wide debate in the scientific community. Concern about this field is present in international studies, such as the one by Baschera et al. [34], seeking to compare realities in various countries, and national, such as the one by Simões et al. [14], reporting that it is necessary to humanize medical education and to deconstruct the image of a profession merely related to social prestige and financial return. In addition to medical education, the reality of the Portuguese NHS has been the subject of discussion in several studies [14,27,33]. These studies results emphasize the importance of a redefinition of Portuguese health policy with regard to improving access and quality of services, NHS economic sustainability, and the promotion of health equity.

The analysis of OR and surgical practice is a priority axis of research at the national and international levels, as improvements will result in gains in health services, satisfaction of health professionals, and overall performance, which will ultimately result in more differentiated healthcare delivery to populations.

## 3. Materials and Methods

### 3.1. Study Design

This is an exploratory quantitative study as it seeks to establish a greater familiarity with the problem under study to make it possible to carry out further and specific future investigations, as referred by Vilelas [40]. The adopted methodology is based on the assumptions of the inductive method, postulating that in an investigation the first step should be based on the exploration of the object of investigation so that, after collecting and analyzing the data, a theory can be elaborated [41]. The objectives that guided this study are to: (i) Describe the main motivations that lead Portuguese surgeons to choose health units for quality surgical practices and (ii) understand how motivations relate to surgeons’ characteristics regarding age, professional experience, intervention area, and sex.

### 3.2. Subjects

This study was conducted with a convenience, non-probabilistic, sample consisting of 99 surgeons of which 67.3% (*n* = 66) were male and 32.7% (*n* = 32) were female. One of the surgeons chose to omit the answer regarding sex. Ages ranged from 37 to 66 years (*M* = 52.4; *Std* = 8.59; *Mdn* = 54). The years of professional experience of surgeons were between 5 and 43 years (*M* = 23.7; *Std* = 8.92, *Mdn* = 25). Table 2 presents the distribution of surgeons by surgical intervention area (omitted = 5).

### 3.3. Instrument

A questionnaire was built to collect data from this study. The survey was divided into two major dimensions. The first refers to the respondents’ socio-demographic and professional component, integrating sex, age, surgical intervention area, and extension of professional experience. A second dimension seeks to explore the motivations of respondents regarding the choice of the healthcare unit to perform surgery, namely through the attribution of the degree of importance, according to a Likert scale, in 13 items, ranging between 1 to 5, and the higher the value obtained, the greater the importance given.

### 3.4. Procedures

This study covered the geographical area of Portugal, and the survey was administered through an electronic form, available on google forms. An invitation to participate was made, and the procedure used to access the group of participants occurred through databases belonging to Portuguese scientific societies, requesting the email contacts of surgeons. In total, 176 surgeons were contacted, corresponding to those accepting to share the email, but only 99 surgeons answered, a response rate of 56.3%. Data were collected between March and April 2018.

In order to ensure that free and truly informed consent was obtained, the scope of the investigation, its objectives and conditions of the study, the procedure to be adapted ensuring complete anonymity and confidentiality of the data provided, and the use for research purposes only, were explained by email. Participants were allowed to post all their questions and clarify them by email with the investigator.

### 3.5. Ethical Approval

All subjects gave their informed consent for inclusion before they participated in the study. The study was conducted in accordance with the Declaration of Helsinki, and the protocol was approved by the Ethics Committee of University Fernando Pessoa (UFP) Porto, Portugal, Project “Conditions for a Differentiated Surgical Practice”, approved in 20 March 2018, no specific reference assigned, date acting as reference ID.

### 3.6. Data Analysis

Statistical analysis of data collected through the questionnaire survey was performed using the IBM^®^ SPSS^®^ Statistics vs.25.0 program [42]. The variables under study were divided into three types: Continuous quantitative variables, namely age and years of professional experience; nominal qualitative variables, namely the intervention area, further categorized into four major areas according to the College of Surgical Specialties of the Portuguese Medical Association [43], participant and sex; and, finally, in ordinal qualitative variables, materialized in the motivations expressed in Likert scales. For the descriptive statistics of continuous quantitative variables, the absolute and relative frequencies were calculated, followed by the means, and their corresponding standard deviations. In the case of ordinal qualitative variables, their distribution was presented according to the median and by the 25th and 75th percentiles.

For ordinal qualitative variables (scale), as it is the case, it is only possible to use non-parametric tests in which the central tendency measure used is the median. In this group of tests, a comparison was made of the distribution of the medians of the motivations between the sexes of the participants through the Mann–Whitney *U* test (two independent samples), in order to understand if there are statistically significant differences. To compare the distribution of medians between the four intervention areas of the participants and their motivations, the Kruskal–Wallis Test (two or more independent samples) was applied. Spearman’s correlation coefficient was applied to obtain the correlations of the variables under study since, i.e., ordinal variables. This test also can also be applied when there is no normality in the distribution, as well as being more sensitive to nonlinear relationships between variables. Initially, the correlations between the variables under study, i.e., sociodemographic and motivations, were calculated and, subsequently, between the three factors extracted from the factor analysis.

Considering the main objective of the study that relates to the surgeons’ motivations underlying the choice of the healthcare unit to perform surgery, it was necessary to find common factors. For this purpose, a Varimax rotation factorial analysis was performed. The mean comparison of the factors extracted from the factor analysis by sex of the surgeons was performed using the Student’s *t*-test for independent samples. In order to verify the existence of mean differences between the surgeons’ intervention areas, the one-way ANOVA was used.

## 4. Results

Table 3 presents the relative frequencies, absolute frequencies, and medians (25th and 75th percentiles) of surgeons’ motivations to perform surgery to understand how respondents’ options are distributed (omitted = 1).

According to data presented in Table 3, all answers are placed in the borderline degrees of importance, i.e., 1 = not important and 5 = very important. It is relevant to note that the item considered as the most important in the decision to choose the healthcare unit to perform the surgery is “Human resources and equipment” with 77.8% of the answers expressed as “very important” (*Mdn* = 5; *P25* = 5; *P75* = 5). At the other extreme, the item “Online visit of facilities” appears as the least important when deciding where to perform surgery with only 3% of responses corresponding to the “very important” option (*Mdn* = 3; *P25* = 2; *P75* = 4).

It is also important to understand the behavior of the variables comparing them by groups. In line with this, Table 4 presents a comparison of the degree of importance attributed to the surgeons’ motivations to perform surgery by sex.

Analyzing Table 4, when comparing surgeons’ motivations by sex, statistically significant differences were identified in relation to “Availability of intensive care for postoperative support” (*p* < 0.05) and “Channels of communication between professionals and hospital structures” (*p* < 0.01), with women being the ones who value these factors the most. A statistically significant difference between sex was not found in any other item.

Table 5 presents a comparison of the degree of importance attributed to surgeons’ motivations to perform surgery by intervention area.

Table 5 highlights that there are no statistically significant differences in motivations to perform surgery between the surgeons’ areas of intervention, except for the item “Partnerships between the different types of health units” (*p* < 0.05) and “Properly addressed indispensable organizational milestones” (*p* < 0.05). Thus, it can be stated that the surgeons who work in the area of “Soft parts surgery” are the ones who most value the quality of interdisciplinary partnerships as a preponderant element in deciding where to perform the surgery (*Mdn* = 5; *P25* = 5; *P75* = 5). On the other hand, the surgeons working in “Oncology and/or breast surgery” give the least importance to this dimension (*Mdn* = 3; *P25* = 2; *P75* = 4). Equally positioned in the degree of importance given to the partnerships are the areas of intervention “General surgery and other intervention areas” and “Digestive surgery” (*Mdn* = 4; *P25* = 3; *P75* = 4). Relating the factor of “Properly addressed indispensable organizational milestones”, surgeons working in the “Soft parts surgery” area are those who value it the most (*Mdn* = 5; *P25* = 5; *P75* = 5), similarly to those working in the “Oncology and/or breast surgery” (*Mdn* = 5; *P25* = 5; *P75* = 5).

After scrutinizing the results of the surgeons’ motivations to perform surgery by sex and by intervention areas, a factor analysis with Varimax rotation allowing the aggregation of the dimensions, i.e., motivation factors, that together explain the variance of the data, is necessary. The Spearman’s correlation coefficient (*r_s_*) was then calculated in order to understand how the variables under analysis are related (Table 6).

Table 6 shows that the importance given to “Properly addressed indispensable organizational milestones” increases when the importance of “Channels of communication between professionals and hospital structures” increases (*r_s_* = 0.66; *p* < 0.01). The same happens between “OR articulation with other sectors of the hospital” and “Channels of communication between professionals and hospital structures” (*r_s_* = 0.59; *p* < 0.01). An equal trend is found between “Properly addressed indispensable organizational milestones” and “Research perspectives” (*r_s_* = 0.58; *p* < 0.01). A positive trend is identified between increasing age and the importance given to “Online visit of facilities” (*r_s_* = 0.32; *p* < 0.01) and between professional experience and “Online visit of facilities” (*r_s_* = 0.30; *p* < 0.01). On the other hand, it was found that the importance attributed to the “Percentage to be paid by surgeon to hospital” decreases as the age of surgeons increases (*r_s_* = −0.30; *p* < 0.01), as well as the professional experience increase too (*r_s_* = −0.23; *p* < 0.05).

Table 7 presents the aggregated factors through Varimax rotation factor analysis. The 13 surgeons’ motivations components of the model, i.e., motivation factors to perform the surgery, were grouped into three factors that together explain 62.1% of the data variability.

In Table 7, the first factor represents the “Available Resources and Infrastructures” and explains, alone, about 28.6% of the data variability and is, therefore, the most preponderant for the decision making of surgeons in choosing the healthcare unit to perform surgery. The “Innovation and Bridges for the Future” factor explains 21.3% of data variability and is the second most important for surgeons. Finally, the third factor “Payment and Geographic Location” explains 12.2% of data variability and is the least influential of surgeons’ decision.

After extracting and analyzing the surgeons’ motivation factors to perform surgery, the associations between them as well as with the age and professional experience factors, were analyzed (Table 8).

According to the Table 7 analysis, weak, although statistically significant, positive relationships were identified between the age of surgeons and the importance attributed to the “Innovation and Bridges to the Future” factor (*r_s_* = 0.28; *p* < 0.01). Similar results were found between the professional experience factor and “Innovation and Bridges to the Future” (*r_s_* = 0.22; *p* < 0.05). Although not statistically significant (ns), weak negative relationships were found between surgeons’ age and the “Available Resources and Infrastructures” (*r_s_* = −0.16; ns) and “Payment and Geographic Location” (*r_s_* = −0.19; ns) factors. Identical relationship between surgeons’ professional experience and the “Available Resources and Infrastructures” (*r_s_* = −0.12; ns) and “Payment and Geographic Location” (*r_s_* = −0.13; ns) factors, was found.

Table 9 presents the results of the Student’s *t*-test by sex of the surgeons, identifying only a statistically significant difference regarding the “Available Resources and Infrastructures” factor (*t* = −2.56; *p* < 0.05). The remaining factors do not present statistically significant differences, either “Innovation and Bridges to the Future” (*t* = −0.36; ns) or “Payment and Geographic Location” (*t* = 0.45; ns). Considering these results, it is possible to conclude female surgeons are those who have a higher average of the “Available Resources and Infrastructures” factor.

The results of the mean differences in factors extracted by surgeon’s intervention area are shown in Table 10.

Through the analysis of Table 10, it can be observed that there are no statistically significant differences between the four major areas of intervention of surgeons, i.e., general surgery and other intervention areas, digestive surgery, oncology and/or breast surgery, and soft parts surgery, regarding the three factors extracted in the factor analysis: “Available Resources and Infrastructures” (*F* = 0.201; *p* > 0.05); “Innovation and Bridges to the Future” (*F* = 0.966; *p* > 0.05); and finally, “Payment and Geographic Location” (*F* = 0.641; *p* > 0.05).

## 5. Discussion

The study results contribute to profiling the motivations that weigh the most in the surgeons’ decisions in relation to the healthcare unit to perform surgery.

The analysis of surgeons’ motivations to perform surgery revealed that, in general, the dimension “Human resources and equipment” is considered the most important for choosing the healthcare unit to perform surgery (77.8%; *Mdn* = 5). This result is in line with the association between age and professional experience (*r_s_* = 0.88; *p* < 0.01), which attaches more importance to dimensions that go far beyond the economic component. This evidence also occurs because the median age of participants is 54 years old, corresponding to surgeons with professional experience. Still at this level, studies already conducted, such as the one by Marjamaa and Kirvela [31], indicate that it is exactly the quality and availability of resources, human or equipment, that most motivate surgeons.

The importance of the payment for performed surgery is of particular relevance to early career professionals. This evidence is in line with the studies already carried out in Portugal and abroad [44], in which medical students are the ones who most value the social prestige and the economic component of the profession (Table 6, *r_s_* = −0.30; *p* < 0.01), such as reported by Baschera et al. [34]. Another interpretation for this result could be related to the fact that with professional experience and years of practice, the expected economic situation of the surgeons, based on known earnings, improves, therefore undermining the importance of the answers given by the older participants.

The results of correlations and factor analysis revealed the interconnection and importance attached to the “Available Resources and Infrastructures” factor. It becomes evident that for surgeons, the quality and organization of OR is crucial for the success of surgical practice, a result already reported by Kähler et al. [45]. There is a diversity of studies on the subject [25,30,35,36,37,38,39,46,47], which corroborate the results presented in this study, pointing to the need for more efficient and effective measures in the OR management.

Considering the exploratory nature of the study, for the advancement of knowledge in the area and future investigations, it is important to present and discuss some results for which there are no comparative studies yet. An example is the comparison that was made of the surgeons’ motivation to perform surgery by sex and intervention area. In the first case, it is the female surgeons who attach the most importance to the “Availability of intensive care for postoperative support” and to the “Channels of communication between professionals and hospital structures”, when compared to male surgeons. When analyzing the surgeons’ motivation by intervention area, it can be stated that at the level of “Partnerships between the different types of health units”, it is the surgeons who work in the area of “Soft parts surgery” that attach the most importance to this dimension. In the dimension “Properly addressed indispensable organizational milestones”, those who most value this are also the surgeons who work in the area of “Soft parts surgery”, observing a follow-up of this trend by the surgeons who work in the area of “Oncology and/or breast surgery”.

Regarding the three major factors extracted, i.e., “Available Resources and Infrastructures”, “Innovation and Bridges to the Future”, and “Payment and Geographic Location” (Table 9), female surgeons attach the most importance (*t* = −2.56; *p* < 0.05) to the resources and infrastructures available in hospitals as a motivating factor to perform surgery. It is also noted that the surgeons’ intervention area (Table 10) should not be considered as a factor determining the motivation for choosing the healthcare unit to perform surgery, i.e., the motivations identified in this study are suggested to be transversal to multiple areas of surgical intervention (*p* > 0.05).

## 6. Conclusions

Managing the surgical process in the OR should be considered as one of the most expensive aspects of all hospital activity, assuming it to be a fundamental but extremely complex challenge. Despite the importance of this task, it is far from gathering indispensable consensus.

The present exploratory approach allows one to conclude that the surgeons’ motivations that underlie the choice of the healthcare unit to perform surgery materialize, in general, in the quality and quantity of human resources and equipment, in the scope of interdisciplinary communication, and an articulation of the OR as a whole integrated in a hospital unit. On the other hand, it is noteworthy that the economic component loses importance, given the results obtained, which is an important factor for the decisions that may result from this study. Also, the geographic location of the hospital does not significantly influence the surgeons’ decision.

It is necessary to continue to deepen this subject, opening the way for broader and comparative studies, with a more representative sample of the population and the inclusion of other domains of knowledge, where the main objective is a continuous improvement of the care provided in the surgical practice.

### Strengths and Limitations

It is important to identify the weaknesses and limitations of the present study, namely in terms of the detailed design of the instrument, the inability to identify active participants, whether they perform functions in the private and/or public sector, and the fact of the difficult treatment of the open question “surgical intervention area”, in general of great complexity. Since the survey was administered online, it conditioned the response rate, which fell short of the extensive number of contacts previously made. To reduce bias, the authors have randomized the order of the questions to avoid “question order bias”. It would have been relevant to consider other socio-economic variables, such as place of education or current salary, considered sensitive data by the Ethics Committee authorizing the study, and thus not allowed. It is also important to acknowledge that the intrinsic motivations of the professional, ethical, and vocational factors were not considered.

Notwithstanding the limitations presented, the study emphasizes the importance of developing and applying similar surveys, so as to promote a culture of knowledge about current surgical practice, helping to improve marketing techniques and proposals aimed to changing deep organizational patterns.

## Figures and Tables

**Table 1:** State of the art relating efficient operating room (OR) management (2000–2018).

Study Identification	Target	Material and Methods	Results
Coordination Challenges in Operating-Room Management: An In-Depth Field Study [30]	Surgeons	Information and communication techniques (ICTs).	Demonstration of the technical importance of ICTs in the OR organization. Information systems provide only a small portion of the information needed to properly manage changing OR programming.
Surgeons also think [19]	Surgeons	Reflections on the teaching and practice of surgery in the different aspects.	Hope for changes in mentality. Change of scientific paradigm. Prevent and care for populations.
Who is responsible for operating room management and how do we measure how well we do it? [31]	Everyone related to surgical activities	Questionnaire applied to various professional OR related groups.	Need to redefine the OR management with a focus on collaboration and communication between different groups. There should be more focus on collaboration and communication among healthcare professionals.
Hospital’s Strategies for Orchestrating selection of physician preference items [32]	Physicians	Analyze hospital strategies for adapting the behavior of physicians and counteracting the power of providers to interfere with professionals’ choices.	Reduce costs by standardizing physicians preferred items. Overcoming barriers.
National Health Plan 2011-2016 [33]	All healthcare related	Definition of a health policy for Portugal.	Continuous improvement of healthcare. Maximization of obtaining health gains in a sustainable manner. Equity and access to healthcare. Hospital network.
Final Report of the Hospital Reform Technical Group [27]	Hospital staff and others	Characterization of the current reality of Portuguese hospitals and reform proposals.	Bases for a consistent hospital reform. Improve access to and quality of healthcare and hospital efficiency. Ensure economic and financial sustainability. Improve governance and performance of hospital professionals. Strengthen the duty of informing citizens.
Scheduling elective surgeries in a Portuguese hospital using a genetic heuristic [25]	Everyone related to surgical activities	Improve the use of available resources and reduce the waiting list for surgery.	Attempt to improve a hospital’s surgical programming using genetic heuristic processes, requiring less staff time than manual procedure.
Are Medical Students Who Want to Become Surgeons Different? An International Cross-Sectional Study [34]	Medical students	Identify the characteristics of medical students aspiring to surgical specialization.	Men students are more prevalent in surgical careers. Search for social and financial prestige.
A surgical scheduling method considering surgeons preferences [35]	Surgeons	Operating time. Surgeons Preferences.	Maximum use of times. Working time as a resource. Surgeon satisfaction improved based on efficient use of time.
Integrated operating room planning and scheduling problem with assistant surgeon dependent surgery durations [36]	Everyone related to surgical activities	Impact of surgeons’ experience on operative times (computational studies).	Expandable algorithm models to improve surgical work scheduling.
Health Systems in Transition [14]	All healthcare related	Contribution to a more effective health policy.	Bases for a more consistent health reform. Increase efficiency and promote financial sustainability of the NHS. Improve efficiency and effectiveness in the NHS, inducing rational use of services and control of spending. Generate additional savings in multiple areas. Decrease public participation in total health expenditure. Reduce inequalities in access to healthcare for the population.
Improving efficiency in preoperative assessment: A pilot study on visit times for preoperative evaluation [37]	Everyone related to surgical activities	Preoperative assessment of patients in a differentiated hospital.	Predictors of the influence of the referred assessment and recognition of its importance to the OR performance.
Satisfied surgeon–patient matching: a model-based method [38]	Surgeons and patients	Algorithm methods for measuring audience preferences. Case study.	Biobjective optimization model for surgeon-patient correspondence and model resolution algorithm. The model consists in maximizing the degree of total satisfaction of surgeons and patients.
Prioritizations of individual surgeons’ patients waiting for elective procedures: A systematic review and future directions [39]	Everyone related to surgical activities	Prioritization program for surgical patients.	Shortage of tools for prioritizing surgical patients.

**Table 2:** Sample characterization by surgical intervention area.

Surgical Intervention Area							
General Surgery and Other Intervention Areas		Digestive Surgery		Oncology and/or Breast Surgery		Soft Parts Surgery	
n	%	n	%	n	%	n	%
72	76.6	14	14.9	7	7.4	1	1.1

**Table 3:** Relative frequencies, absolute frequencies, and medians (25th and 75th percentiles) of surgeons’ motivations to perform surgery.

Motivation Factors	Scale					*Mdn* (*P25–P75*)
	1	2	3	4	5	
	n (%)					
Human resources and equipment	1 (1.0)	1 (1.0)	0 (0.0)	20 (20.2)	77 (77.8)	5 (5 – 5)
Being a modern hospital	1 (1.0)	3 (3.0)	22 (22.2)	47 (47.5)	26 (26.3)	4 (3 – 5)
Percentage to be paid by surgeon to hospital	7 (7.1)	4 (4.0)	21 (21.2)	44 (44.4)	23 (23.2)	4 (3 – 4)
Properly addressed indispensable organizational milestones*	2 (2.0)	2 (2.0)	10 (10.2)	44 (44.9)	40 (40.8)	4 (4 – 5)
Partnerships between the different types of health units	1 (1.0)	9 (9.1)	22 (22.2)	51 (51.5)	16 (16.2)	4 (3 – 4)
Availability of intensive care for postoperative support	1 (1.0)	1 (1.0)	4 (4.0)	29 (29.3)	64 (64.6)	5 (4 – 5)
OR articulation with other sectors of the hospital	1 (1.0)	1 (1.0)	7 (7.1)	42 (42.4)	48 (48.5)	4 (4 – 5)
Research perspectives	6 (6.1)	10 (10.1)	27 (27.3)	39 (39.4)	17 (17.2)	4 (3 – 4)
Imaging laboratory support	0 (0.0)	1 (1.0)	2 (2.0)	29 (29.3)	67 (67.7)	5 (4 – 5)
Channels of communication between professionals and hospital structures	1 (1.0)	1 (1.0)	10 (10.1)	45 (45.5)	42 (42.4)	4 (4 – 5)
Online visit of facilities	16 (16.2)	13 (13.1)	37 (37.4)	30 (30.3)	3 (3.0)	3 (2 – 4)
Influence of facilities	3 (3.0)	1 (1.0)	4 (4.0)	59 (59.6)	32 (32.3)	4 (4 – 5)
Geographic location of hospital	4 (4.0)	6 (6.1)	23 (23.2)	48 (48.5)	18 (18.2)	4 (3 – 4)

Scale: 1 = not important to 5 = very important.

**Table 4:** Medians (25th and 75th percentiles) of surgeons’ motivations to perform surgery and the corresponding mean differences by sex (Mann–Whitney *U* Test for independent samples).

Motivation Factors	Male	Female
	*Mdn* (*P25* – *P75*)	
Human resources and equipment	5 (4 – 5)	5 (5 – 5)
	0.24	
Being a modern hospital	4 (3 – 5)	4 (3 – 4.75)
	0.71	
Percentage to be paid by surgeon to hospital	4 (3 – 5)	4 (3 – 4)
	0.673	
Properly addressed indispensable organizational milestones	4 (4 – 5)	5 (4 – 5)
	0.06	
Partnerships between the different types of health units	4 (3 – 5)	4 (4 – 4)
	0.23	
Availability of intensive care for postoperative support	5 (4 – 5)	5 (5 – 5)
	**0.02**	
OR articulation with other sectors of the hospital	4 (4 – 5)	5 (4 – 5)
	0.09	
Research perspectives	4 (3 – 4)	4 (3 – 4)
	0.41	
Imaging laboratory support	5 (4 – 5)	5 (4.25 – 5)
	0.22	
Channels of communication between professionals and hospital structures	4 (4 – 5)	5 (4 – 5)
	**0.00**	
Online visit of facilities	3 (2 – 4)	3 (2.25 – 4)
	0.78	
Influence of facilities	4 (4 – 5)	4 (4 – 5)
	0.69	
Geographic location of hospital	4 (3 – 4)	4 (3 – 4.75)
	0.70	
Scale: 1 = not important to 5 = very important; significant differences are presented in bold (*p* < 0.05)		

**Table 5:** Medians (25th and 75th percentiles) of surgeons’ motivations to perform surgery and the corresponding mean differences by surgical intervention area (Kruskal–Wallis Test for independent samples).

Motivation Factors	Surgical Intervention Area			
	General Surgery and Other Intervention Areas	Digestive Surgery	Oncology and/or Breast Surgery	Soft Parts Surgery
	*Mdn* (*P25* – *P75*)			
Human resources and equipment	5 (4.25 – 5)	5 (5 – 5)	5 (4 – 5)	5 (5 – 5)
	0.82			
Being a modern hospital	4 (3 – 4.75)	4 (3 – 5)	4 (4 – 5)	5 (5 – 5)
	0.38			
Percentage to be paid by surgeon to hospital	4 (3 – 4)	4 (2.75 – 5)	4 (3 – 5)	5 (5 –5)
	0.53			
Properly addressed indispensable organizational milestones	4 (4 – 5)	4 (4 – 5)	5 (5 – 5)	5 (5 – 5)
	0.05			
Partnerships between the different types of health units	4 (3 – 4)	4 (3 – 4)	3 (2 – 4)	5 (5 – 5)
	**0.04**			
Availability of intensive care for postoperative support	5 (4 – 5)	5 (4.75 – 5)	4 (4 – 5)	5 (5 – 5)
	0.47			
OR articulation with other sectors of the hospital	4 (4 – 5)	5 (4 – 5)	5 (4 – 5)	5 (5 – 5)
	0.49			
Research perspectives	3.5 (3 – 4)	4 (3 – 5)	4 (4 – 4)	5 (5 – 5)
	0.07			
Imaging laboratory support	5 (4 – 5)	5 (4 – 5)	5 (5 – 5)	5 (5 – 5)
	0.64			
Channels of communication between professionals and hospital structures	4 (4 – 5)	4 (4 – 5)	5 (5 – 5)	5 (5 – 5)
	0.08			
Online visit of facilities	3 (2 – 4)	3 (2 – 4)	3 (3 –4)	4 (4 – 4)
	0.56			
Influence of facilities	4 (4 – 5)	4 (4 – 5)	4 (4 – 5)	5 (5 – 5)
	0.59			
Geographic location of hospital	4 (3 – 4)	4 (4 – 5)	4 (4 – 5)	4 (4 – 4)
	0.16			

Scale: 1 = not important to 5 = very important; significant differences are presented in bold (*p* < 0.05)

**Table 6:** Spearman’s correlation coefficient between the variables under study.

Motivation Factors	1	2	3	4	5	6	7	8	9	10	11	12	13	14	15
**1. Age**	1														
**2. Professional experience**	0.88 **	1													
**3. Human resources and equipment**	−0.16	−0.16	1												
**4. Being a modern hospital**	0.02	−0.04	0.35 **	1											
**5. Percentage to be paid by surgeon to hospital**	−0.30 *	−0.23 *	0.19	0.20 *	1										
**6. Properly addressed indispensable organizational milestones**	0.14	0.08	0.30 **	0.38 **	−0.03	1									
**7. Partnerships between the different types of health units**	0.07	0.02	0.19	0.30 **	0.09	0.38 **	1								
**8. Availability of intensive care for postoperative support**	−0.01	−0.0	0.47 **	0.17	0.04	0.26 **	0.14	1							
**9. OR articulation with other sectors of the hospital**	−0.06	0.01	0.45 **	0.32 **	0.13	0.47 **	0.36 **	0.47 **	1						
**10. Research perspectives**	0.14	0.12	0.27 **	0.36 **	−0.04	0.58 **	0.37 **	0.33 **	0.42 **	1					
**11. Imaging laboratory support**	−0.02	−0.01	0.48 **	0.35 **	0.12	0.44 **	0.19	0.26 **	0.35 **	0.31 **	1				
**12. Channels of communication between professionals and hospital structures**	0.07	0.09	0.30 **	0.33 **	0.16	0.66 **	0.35 **	0.37 **	0.60 **	0.52 **	0.46 **	1			
**13. Online visit of facilities**	0.33 **	0.30 **	0.08	0.26 *	0.07	0.38 **	0.29 **	0.14	0.19 *	0.38 **	0.26 *	0.43 **	1		
**14. Influence of facilities**	−0.09	−0.11	0.39 **	0.53 **	0.16	0.37 **	0.26 **	0.25 *	0.41 **	0.31 **	0.34 **	0.41 **	0.16	1	
**15. Geographic location of hospital**	−0.07	−0.04	0.15	0.27 **	0.35 **	0.17	0.06	0.05	0.20 *	0.19	0.15	0.19	0.09	0.17	1

* Significant correlation at *p* < 0.05 level; ** significant correlation at *p* < 0.01 level.

**Table 7:** Varimax rotation factor analysis of the surgeons’ motivations to perform surgery.

Motivation Factors	Factor Loads		
**Available Resources and Infrastructures|28.6%**	I	II	III
Human resources and equipment	0.877		
Properly addressed indispensable organizational milestones	0.532		
Availability of intensive care for postoperative support	0.849		
OR articulation with other sectors of the hospital	0.741		
Imaging laboratory support	0.667		
Channels of communication between professionals and hospital structures	0.644		
Influence of facilities	0.570		
**Innovation and Bridges to the Future|21.3%**			
Being a modern hospital		0.517	
Partnerships between the different types of health units		0.699	
Research perspectives		0.695	
Online visit of facilities		0.741	
**Payment and Geographic Location|12.2%**			
Percentage to be paid by surgeon to hospital			0.816
Geographic location of hospital			0.749

Note: Extraction method: Principal components. Varimax rotation with Kaiser normalization. Extraction criterion: Eigenvalues higher than one. Total variance explained by extracted components: 62.1%; *Kaiser–Meyer–Olkin* = 0.86; Bartlett’s test: *χ*^2^ = 4765.04, *p* < 0.05; measures sampling adequacy > 0.5; global Cronbach’s alpha (α): α = 0.89.

**Table 8:** Spearman’s correlation coefficient between the variables age and professional experience and the three components extracted from the factor analysis, addressing resources and infrastructures, innovation, and payment and location.

Factors	1	2	3	4	5
**1. Age**	1				
**2. Professional Experience**	0.88 **	1			
**3. Available Resources and Infrastructures**	−0.16	−0.12	1		
**4. Innovation and Bridges to the Future**	0.28 **	0.22 *	−0.06	1	
**5. Payment and Geographic Location**	−0.19	−0.13	−0.09	0.04	1

* Significant correlation at *p* < 0.05 level; ** significant correlation at *p* < 0.01 level.

**Table 9:** Means comparison of the three factors extracted from factor analysis by sex of surgeons using Student’s *t*-test for independent samples, addressing resources and infrastructures, innovation, and payment and location.

Factors	Sex	*n*	*M*	*Std*	*t*	*p*
**Available Resources and Infrastructures**	Male	65	−0.16	1.09	−2.56	0.012 *
	Female	32	0.31	0.70		
**Innovation and Bridges to the Future**	Male	65	0.00	0.94	−0.36	0.721
	Female	32	0.08	1.06		
**Payment and Geographic Location**	Male	65	0.03	1.03	0.45	0.652
	Female	32	−0.07	0.97		

* Significant difference at *p* < 0.05 level.

**Table 10:** Mean factor comparison by surgeon’s intervention area using one-way ANOVA, addressing resources and infrastructures, innovation, and payment and location.

Factors	*F*	*p*
**Available Resources and Infrastructures**	0.201	0.896
**Innovation and Bridges to the Future**	0.966	0.413
**Payment and Geographic Location**	0.641	0.591
